# Extra-anatomic left subclavian artery bypass patency in frozen elephant trunk surgery

**DOI:** 10.1016/j.xjtc.2025.01.025

**Published:** 2025-02-11

**Authors:** Leon Mattern, Philipp Pfeiffer, Karen Wittemann, Edoardo Zancanaro, Chris Probst, Ahmed Ghazy, Hendrik Treede, Daniel-Sebastian Dohle

**Affiliations:** Department of Cardiac and Vascular Surgery, University Medical Center Mainz, Mainz, Germany

**Keywords:** frozen elephant trunk, aortic arch surgery, left subclavian artery bypass, ischemic burden

## Abstract

**Objective:**

To explore the advantages and consequences of using an extra-anatomic Dacron bypass in frozen elephant trunk surgery for fast and secure left subclavian artery (LSA) reimplantation.

**Methods:**

Between June 2017 and June 2023, 195 patients were treated using an LSA bypass. All postoperative imaging was reviewed to assess the patency of the bypass grafts. If the LSA bypass was not patent, symptoms of complications and their management were evaluated. Time-to-event analysis was performed to assess bypass patency and time to thrombosis.

**Results:**

Out of 195 LSA bypasses, 183 remained patent during follow-up, for a 5-year patency rate of 91.4%. Prolonged cardiopulmonary bypass duration was associated with poorer graft patency. Eight of the 12 patients with a thrombosed LSA bypass were asymptomatic. The most common complication of thrombosed bypass was subclavian steal syndrome. Surgical revision was necessary in 2 of the 4 symptomatic patients. All cases of thrombosed LSA bypass occurred within the first 15 months.

**Conclusions:**

LSA bypass in frozen elephant trunk surgery is a fast and safe technique for supra-aortic artery reimplantation. Bypass thrombosis is rare and often does not require surgical intervention.

**Figure:**
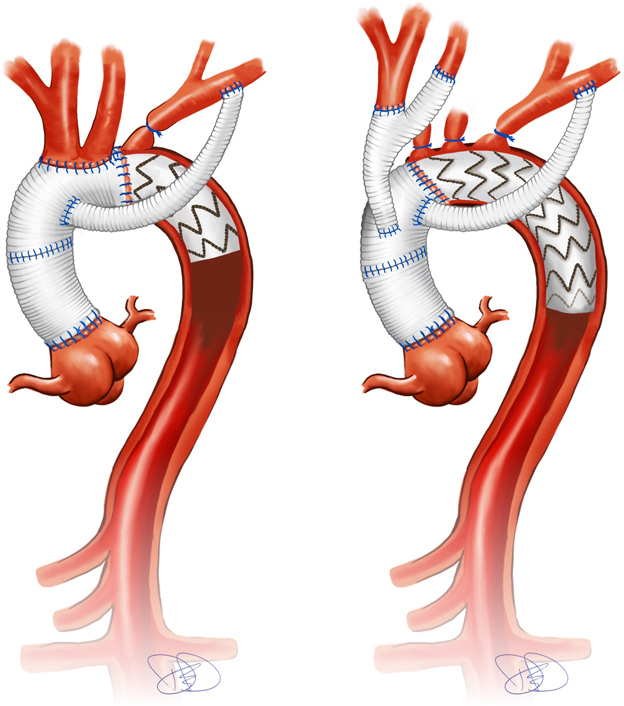
Extra-anatomic LSA management in zones 0 and 2.

**Central Message**

Left subclavian artery bypass in frozen elephant trunk surgery offers a reliable, time-efficient, and durable technique for reimplantation. Complications are rare and easily manageable.

**Perspective**

Safe and fast reimplantation of supra-aortic vessels remains a key question in aortic arch surgery with frozen elephant trunk. With our strategy of left subclavian artery bypass implementation before or after deep hypothermic cardiac arrest, the patient's ischemic burden can be limited. This safe, quick, and sturdy technique enriches the surgical armamentarium.

Despite recent advances, surgical treatment of aortic arch disease with frozen elephant trunk (FET) still carries a considerable risk for death and morbidity due to stroke, respiratory, and renal failure.^1^, ^2^, ^3^ Nevertheless, the FET is increasingly being used to treat acute and chronic dissections, as well as large thoracic aortic aneurysms, with excellent long-term results.^1^^,^^4^ However, this type of surgery, involving hypothermic circulatory arrest and extended cardiopulmonary bypass (CPB) times, poses a significant strain on the patient. Consequently, concepts aimed at reducing the ischemic burden have been developed. These include proximalization of the distal anastomosis into zone 2, as described by Detter and colleagues in 2019^5^ and by Tsagakis and colleagues in 2021.^6^ Proximalization avoids left subclavian artery (LSA) management during hypothermic circulatory arrest (DHCA) or cardiac ischemia. Instead, LSA management is addressed either at the beginning or the end of the surgery, reducing DHCA times and cardiac ischemia times.^5^, ^6^, ^7^, ^8^ Alternative concepts, including stent-bridging of the supra-aortic vessel anastomoses, also aim to reduce the DHCA time by accelerating the carotid and LSA anastomosis.^9^, ^10^, ^11^

At our institution, we routinely close the LSA and establish an extra-anatomic aortoaxillary bypass through the second intercostal space using a Dacron graft. One concern expressed about the strategy of using extra-anatomic LSA management is the potentially limited long-term patency of the extra-anatomic bypass. There is little data in the literature regarding the patency rates of LSA bypasses, and particularly no clear evidence on the clinical significance of a bypass occlusion or how best to manage it.^12^^,^^13^ Therefore, the present study aimed to investigate the long-term patency rates of the extra-anatomic LSA bypasses performed in our FET cohort.

## Patients and Methods

### Ethical Statement

These data, combined with medical records, were approved for use in human subject research by the Institutional Ethics Committee of the Rheinland-Pfalz Medical Association (2018-13574), with patient consent waived owing to the use of retrospective, anonymized data.

### Patient Population

A total of 221 consecutive patients underwent aortic arch replacement with FET at our institution between June 2017 and June 2023. Because this was a single-center study, all operations were performed at University Medical Center Mainz, and no external cases were included. Patients with missing imaging data (n = 10) and those with other methods of LSA revascularization (n = 16) were excluded.

Only patients treated with LSA rerouting who had sufficient postoperative imaging were included in this study (n = 195). Patients with missing imaging (n = 10) and those with other methods of LSA revascularization (n = 16) were excluded.

### Operative Technique

Invasive monitoring included radial and femoral arterial lines and a central venous catheter. We used bilateral near-infrared spectroscopy for cerebral oxygenation monitoring and measured bladder temperature to track core body temperature. Cannulation was usually performed with an end-to-side anastomosis of an 8-mm graft to the right subclavian artery (RSA; 73%); alternative perfusion strategies included LSA access (12%) and direct true lumen cannulation (14%). Extracorporeal circulation was established early to achieve fast cooling to approximately 26 °C, to begin the procedure with the arch. After completing the distal anastomosis of the FET and either reimplanting or debranching the supra-aortic vessels, we resumed perfusion and began gradual rewarming. Once distal and cerebral perfusion was restored, we performed procedures on the aortic root and ascending aorta during the rewarming phase.

The extra-anatomic bypass of the LSA was carried out during the myocardial reperfusion after cross-clamp removal with the heart beating and all other organs except the left arm perfused. To achieve this, we exposed the LSA through an infraclavicular incision. We bluntly divided the pectoralis major muscle and pushed the anterior edge of the pectoralis minor muscle laterally. We then isolated the axillary artery and made a longitudinal incision. We sewed a 7- or 8-mm, 30-cm-long graft to the axillary artery in an end-to-side fashion with a running 6/0 or 7/0 polypropylene suture. The choice of graft size (7 or 8 mm) was not predetermined; rather, the selected size corresponded most closely to the diameter of the vessel, ensuring an appropriate match for the patient's anatomy.

After flushing the graft retrogradely with heparinized blood, we tied it off. We identified the second intercostal space above the subclavian vein by gently probing it with a finger, then passed a clamp and tape through it into the mediastinum. We pulled the graft into the mediastinum through this space, ensuring a straight, kink-free alignment. After measuring the appropriate length of the graft to allow for chest closure, we cut it accordingly and sutured it end-to-end with 5-0 polypropylene suture to the lateral branch of the FET prosthesis.

Weaning from CPB was initiated once sufficient reperfusion was achieved and a target temperature of 36.5 °C was reached. The heart-lung machine was subsequently discontinued, and decannulation was performed. Hemostasis was ensured by administering protamine in accordance with the initial heparin dose, substituting coagulation factors, and proceeding with the closure of the sternum and surgical wound (Video 1).

### Data Curation

Data were collected prospectively from our institutional aortic surgery database and analyzed retrospectively. Patient history, comorbidities, and previous surgical interventions were gathered reviewing existing medical records. Operative details and perfusion times were assembled by surgeon's report and perfusionist protocol. Aortic arch anomalies and preoperative dissections status were determined by computed tomography angiography (CTA).

Overall survival, symptoms, and patency of the extra-anatomic LSAB were assessed during the routine follow-up with CTA at discharge, after 6 months, and annually or longer intervals thereafter. The most recent available CTA was used to evaluate the patency of the extra-anatomic LSA bypass. If the whole graft from the aortic orifice to the LSA orifice showed contrast, the LSAB was deemed open. In cases of thrombosed LSAB, follow-up imaging was revisited to discover at which time the graft was last perfused. Additionally, the symptoms of the patient with thrombosed LSAB and the consequent treatment were evaluated.

### Data Analysis

Analysis was conducted with Prism version 10.1.1 for MacOS (GraphPad Software; www.graphpad.com). Categorial variables are presented as count and percentage and were compared using the Fisher exact test owing to the limited sample size of the subgroup with thrombosed aortoaxillary bypass. For continuous variables, normal distribution was tested with the Shapiro-Wilk test. If the assumption held, the 2-sided Student *t* test was used; if not, the Mann-Whitney *U* test was applied. Kaplan-Meier analysis was used for time-to-event and survival analysis. The statistical significance of differences between survival times and time-to-event analysis was checked by applying the log-rank method. Results were considered statistically significant at *P* < .05.

## Results

### Preoperative Details and Comorbidities

Indication for surgery was acute aortic dissection (AAD) in 65.1% of patients, chronic aortic dissection (CAD) in 23.1%, and thoracic aortic aneurysm (TAA) in 11.8%. 65 operations (33.3%) were performed in patients who had undergone previous cardiac or aortic surgery. The mean patient age at surgery was 61 ± 11 years, and the cohort was predominantly male (71.3%). The most common comorbidities were hypertension (80.0%), dyslipidemia (38.5%), and history of nicotine use (26.7%). There were no significant differences in preoperative variables between the patent LSAB and thrombosed LSAB groups (Table 1).

**Table 1:** Preoperative patient characteristic of patients with patent and thrombosed LSAB

Characteristic	Total (N = 195)	Patent LSAB (N = 183)	Thrombosed LSAB (N = 12)	*P* value
Age at surgery, y, mean ± SD	61.3 ± 10.5	61.4 ± 10.4	59.2 ± 11.5	.25
Male sex, n (%)	139 (71.3)	130 (71.0)	9 (75.0)	>.99
BMI, mean ± SD	27.8 ± 5.4	27.9 ± 5.5	26.4 ± 2.9	.50
Previous cardiac or aortic surgery, n (%)	65 (33.3)	62 (33.9)	3 (25.0)	.75
Disease, n (%)				
AAD	127 (65.1)	119 (65.0)	8 (66.7)	>.99
CAD	45 (23.1)	44 (24.0)	1 (8.3)	.30
TAA	23 (11.8)	20 (10.9)	3 (25.0)	.15
Penn classification for AAD (N = 127), n (%)				
A	48 (37.8)	44 (37.0)	4 (50.0)	.48
AB	51 (40.2)	49 (41.2)	2 (25.0)	.47
AC	8 (6.3)	8 (6.7)	0 (0.0)	>.99
ABC	20 (15.7)	18 (15.1)	2 (25.0)	.61
Aortic arch anomalies, n (%)				
Bovine arch	33 (16.9)	30 (16.4)	3 (25.0)	.43
Aberrant right subclavian artery	5 (2.6)	5 (2.7)	0 (0.0)	>.99
Isolated vertebral artery	9 (4.6)	9 (4.9)	0 (0.0)	>.99
Comorbidities, n (%)				
COPD	18 (9.2)	17 (9.3)	1 (8.3)	>.99
History of nicotine use	52 (26.7)	48 (26.2)	4 (33.3)	.74
Hypertension	156 (80.0)	147 (80.3)	9 (75.0)	.71
Diabetes	10 (5.1)	9 (4.9)	1 (8.3)	.48
Dyslipidemia	75 (38.5)	70 (38.3)	5 (41.7)	>.99
Kidney disease	17 (8.7)	17 (9.3)	0 (0.0)	.60
Coronary artery disease	29 (14.9)	27 (14.8)	2 (16.7)	.69
Supra-aortic vessel dissection in AAD and CAD (N = 172), n (%)				
Innominate artery	103 (59.9)	98 (53.6)	5 (41.7)	>.99
Right common carotid artery	56 (32.6)	53 (29.0)	3 (25.0)	>.99
RSA	28 (16.3)	25 (13.7)	3 (25.0)	.16
Left common carotid artery	81 (47.1)	76 (41.5)	5 (41.7)	.74
LSA	85 (49.4)	80 (43.7)	5 (41.7)	.75

*LSAB*, Left subclavian artery bypass; *BMI*, body mass index; *AAD*, acute aortic dissection; *CAD*, chronic aortic dissection; *TAA*, thoracic aortic aneurysm; *COPD*, chronic obstructive pulmonary disease; *RSA*, right subclavian artery; *LSA*, left subclavian artery.

### Intraoperative Details

Cannulation of the RSA via an 8-mm prosthesis was the primary approach (73.8% of patients). If RSA cannulation was not feasible because of RSA dissection, lusorial artery, or patient factors, then LSA cannulation (12.3%) or direct true lumen cannulation (DTLC; 13.8%) was used.

Hypothermic circulatory arrest was performed at 24.5 ± 2.2 °C. Unilateral cerebral perfusion was applied in 57.4% of cases; bilateral perfusion via an extra pump was required in 40.5% due to decreased near-infrared spectroscopy values or debranching.; Distal anastomosis was placed in zone 2 in 70.3% of cases. The innominate artery and left common carotid artery were reimplanted using the island technique in 76.6% of cases or via debranching in 23.4%. Zone 0 was used in 29.7%, with separate debranching of all supra-aortic vessels. For LSA reimplantation via bypass, a 7-mm Dacron graft was used in 76.9%. Additional details are provided in Table 2.

**Table 2:** Intraoperative details for patients with patent and thrombosed LSAB

Operative details	Total (N = 195)	Patent LSAB (N = 183)	Thrombosed LSAB (N = 12)	*P* value
Cannulation site, n (%)				
RSA	144 (73.8)	136 (74.3)	8 (66.7)	.52
LSA	24 (12.3)	22 (12.0)	2 (16.7)	.65
DTLC	27 (13.8)	25 (13.7)	2 (16.7)	.67
Perfusion strategy, n (%)				
Unilateral	112 (57.4)	107 (58.5)	5 (41.7)	.37
Bilateral	79 (40.5)	72 (39.3)	7 (58.3)	.23
Retrograde	4 (2.1)	4 (2.2)	0 (0.0)	>.99
Perfusion details				
CPB time, min, mean ± SD	252.7 ± 50.3	251.8 ± 51.2	266.5 ± 24.0	.18
Cross-clamp time, min, mean ± SD	139.1 ± 42.5	137.4 ± 42.3	164.2 ± 38.8	**.02**
Temperature, °C, mean ± SD	24.5 ± 2.2	24.5 ± 2.2	24.6 ± 2.0	>.99
Cerebral perfusion time, min, mean ± SD	53.0 ± 14.9	52.9 ± 15.2	54.7 ± 8.6	.37
Distal ischemia time, min, mean ± SD	29.2 ± 12.8	29.0 ± 12.6	31.8 ± 15.8	.69
Distal anastomosis, n (%)				
Zone 0	58 (29.7)	54 (29.5)	4 (33.3)	.49
Zone 2	137 (70.3)	129 (69.9)	8 (66.7)	.76
Supra-aortic branch management, n (%)				
Island	105 (53.8)	99 (54.1)	6 (50.0)	>.99
Debranching	90 (46.2)	84 (45.9)	6 (50.0)	>.99
LSAB graft size, n (%)				
6 mm	2 (1.0)	2 (1.1)	0 (0.0)	>.99
7 mm	150 (76.9)	140 (76.5)	10 (83.3)	.74
8 mm	36 (18.5)	35 (19.1)	1 (8.3)	.70
10 mm	7 (3.6)	6 (3.3)	1 (8.3)	.36
Concomitant procedures, n (%)				
CABG	13 (6.7)	11 (6.0)	2 (16.7)	.19
Aortic valve intervention				
Repair	86 (44.1)	82 (44.8)	4 (33.3)	.55
Replacement	29 (14.9)	25 (13.7)	4 (33.3)	.08
Aortic root intervention				
Bentall procedure	20 (10.3)	17 (9.3)	3 (25.0)	.11
David procedure	11 (5.6)	10 (5.5)	1 (8.3)	.51
Isolated sinus replacement	14 (7.2)	13 (7.1)	1 (8.3)	.60
Outcome				
In-hospital mortality, n (%)	14 (7.2)	14 (7.7)	0 (0)	.32
1-y survival, %	88	87	92	.91
2-y survival, %	85	85	73	.91
Stroke, n (%)	17 (8.7)	17 (9.3)	0 (0)	.39
Thoracotomy, n (%)	16 (8.2)	16 (9.4)	0 (0)	.29

Bold values indicate statistical significance was asumed for *P* < .05. *LSAB*, Left subclavian artery bypass; *RSA*, right subclavian artery; *LSA*, left subclavian artery; *DTLC*, direct true lumen cannulation; *CPB*, cardiopulmonary bypass; *CABG*, coronary artery bypass grafting.

### LSAB Patency

**Figure 1:**
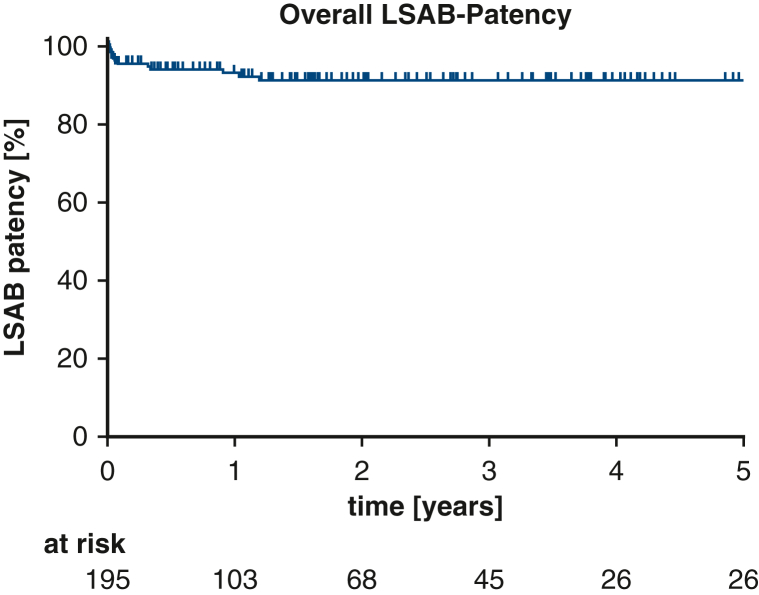
Overall left subclavian artery bypass (*LSAB*) patency.

**Figure 2:**
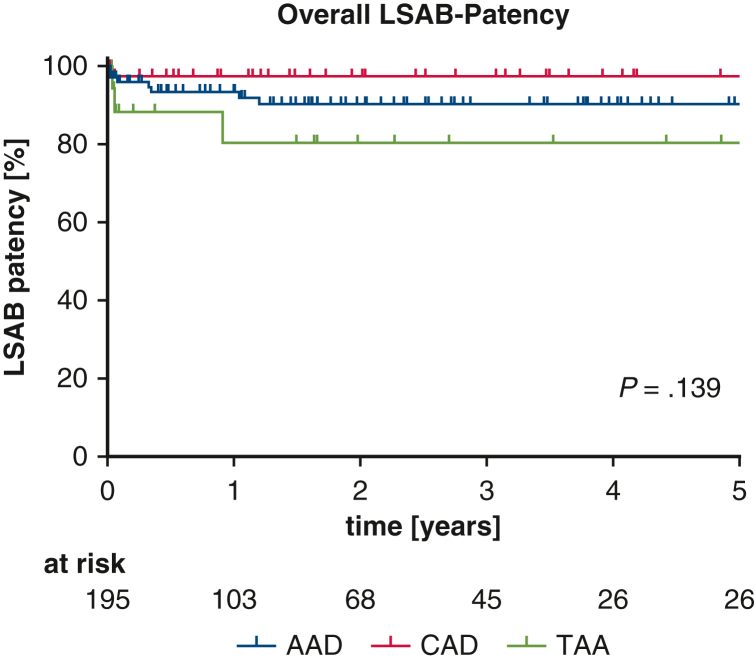
Left subclavian artery bypass (*LSAB*) patency for different indications for surgery. *AAD*, Acute aortic dissection; *CAD*, chronic aortic dissection; *TAA*, thoracic aortic aneurysm.

Among the 195 patients receiving extra-anatomic LSAB, 12 developed thrombosis during follow-up, all within 436 days postoperatively. Five-year patency was 91.4% overall, with no significant differences between island (92.6%) and debranching (89.0%) techniques (Figure 1). Patency rates by indication were 97.7% for CAD, 90.5% for AAD, and 80.5% for TAA (*P* = .1390) (Figure 2). No associations were observed between patency and supra-aortic vessel management, comorbidities, cannulation/perfusion strategies, concomitant procedures, or graft size. Only shorter cross-clamp times correlated significantly with improved patency rates (*P* = .0236).

### Complications and Management of LSAB Occlusion

**Figure 3:**
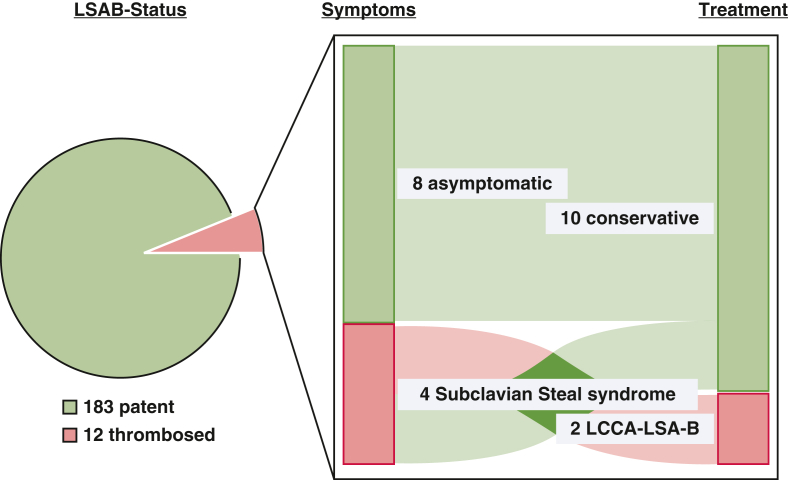
Symptoms and treatment of thrombosed left subclavian artery bypass (*LSAB*). *LCCA–LSAB*, Left common carotid artery to left subclavian artery bypass.

Thrombosis of the extra-anatomic LSAB occurred in 12 patients, 6 of whom remained asymptomatic and required no intervention. One patient presented with a seroma at the graft–LSA anastomosis, which was managed conservatively. Four patients developed subclavian steal syndrome necessitating extrathoracic left common carotid artery–LSA bypass; 2 of these patients declined reintervention. Overall, 2 of 12 patients (16.7%) required surgical revision (Figure 3). All carotid subclavian bypass procedures were performed without perioperative events.

### Follow-Up and Survival

**Figure 4:**
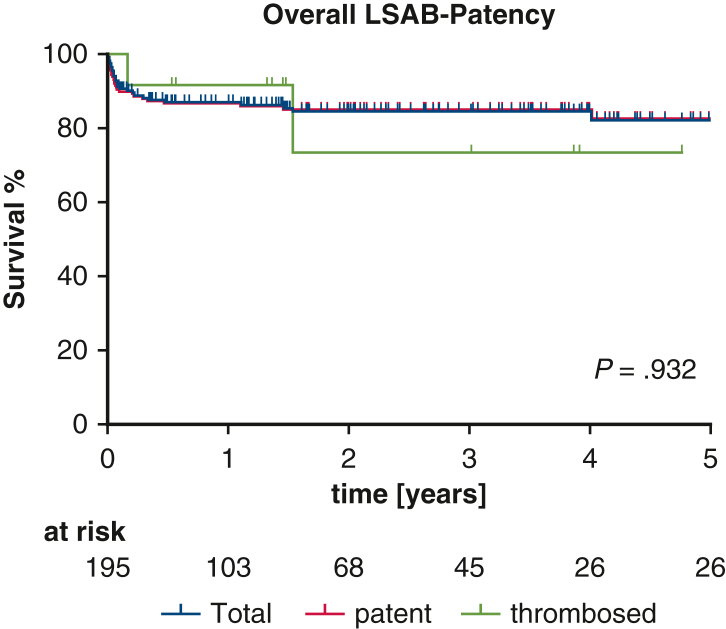
Survival of patients with patent and thrombosed left subclavian artery bypass (*LSAB*).

In the selected cohort, excluding in-hospital mortality, the 5-year survival rate was 82.2% (Figure 4), with a mean follow-up of 2.0 ± 1.7 years. A trend toward improved survival was noted among patients with patent postcapillary bypasses compared to those with thrombosed grafts, but the small number of occlusions limits our ability to draw definitive conclusions.

## Discussion

Although the term “ischemic burden” refers to cardiac ischemia and encompasses the duration, extent, severity, and frequency of ischemic episodes, it remains challenging to define precisely.^14^ In the context of aortic surgery, prefinalization concepts ultimately aim to reduce the ischemic burden on the entire body. This reduction is particularly crucial in cases of dissection with shock, MA perfusion, or both owing to the already existing ischemic burden and the reperfusion initiated by the CPB connection.

In addition, the approach described here significantly reduces operative time for both the distal FET anastomosis and LSA management. The ischemic burden is reduced by proximal zing and simplifying the distal anastomosis. Performing the distal anastomosis in zone 2 or 0 places it closer to the surgeon, facilitating a technically simpler and therefore more rapid procedure. However, the most significant reduction in distal ischemia time likely is achieved by avoiding management of the LSA during circulatory arrest. When managed anatomically, the limited exposure of the LSA is typically the most challenging and time-consuming step during DHCA.

The concept that we and others describe involves the use of extra-anatomic bypass to the LSA, which can be constructed either before sternotomy or after reopening the aortic clamp and cardiac ischemia. In both cases, LSA management does not occur during cardiac or distal ischemia time. Our present results show that this approach yields comparable outcomes to those reported by Detter and colleagues^5^ and by Tasaki's and colleagues,^6^^,^^7^ as well as others, regarding CPB time (252 minutes vs 232 minutes vs 243 minutes), cross-clamp time (139 minutes vs 114 minutes vs 116 minutes), and DHCA times (29 minutes vs 42 minutes vs 32 minutes).

The agreement of our results with those from groups that also use extra-anatomic LSA management—specifically regarding DHCA, cross-clamp, and CPB times—confirms the reproducibility of this concept. Other groups operating in zone 3 and performing a conventional anatomic LSA reconstruction have report median times of 55 minutes for DHCA, 126 minutes for cross-clamp, and 229 minutes for CPB.^15^ This highlights the time-saving potential, particularly during DHCA.

Other concepts that favor anatomic management of the LSA also avoid intrathoracic anastomosis of the LSA and attempt to limit distal ischemia by using a stent instead of an anastomosis.^9^^,^^11^ However, these methods result in longer distal ischemia times, 60 minutes compared to only 30 minutes.^10^ The patency rates of stents and extra-anatomic bypass are similar (95% vs 98%).^10^

Despite the benefits of extra-anatomic LSAB, a commonly cited drawback is a concern about long-term durability. In our cohort, extra-anatomic LSAB has had good durability, and thrombosed bypasses have not resulted in significant clinical issues. Among the 12 patients who experienced thrombosis, 8 were asymptomatic and did not require further treatment. Only 2 patients required surgical reintervention, which was successfully managed with no perioperative complications.

Overall, our study indicates that the strategy of using an extra-anatomic LSAB is a safe and effective method. It provides good short-term outcomes by reducing the ischemic burden and demonstrates excellent long-term results, particularly in terms of the patency of the bypass.

### Limitations

The signiﬁcance of our results is limited by the study's retrospective design. Because this was a single-center experience with a limited number of different surgeons, the external validity of our findings is unknown. Furthermore, although we had a high rate of patency, we were not able to determine the causes of LSAB thrombosis. Verifying the significance of our findings will require collection and analysis of more data on a multicenter basis.

## Conclusions

Our findings support extra-anatomic LSAB as a safe, reliable technique with favorable short-term and long-term outcomes. This approach effectively reduces the ischemic burden during FET surgery and has a high patency rate, thereby addressing concerns about the long-term durability of these bypasses.

## Conflict of Interest Statement

Dr Pfeiffer reported receipt of educational grants for the 2022 and 2024 DGTHG Conferences and for the 36th EACTS Annual Meeting in 2022. All other authors reported no conflicts of interest.

The *Journal* policy requires editors and reviewers to disclose conflicts of interest and to decline handling or reviewing manuscripts for which they may have a conflict of interest. The editors and reviewers of this article have no conflicts of interest.

## Supplementary Data

Video 1 Demonstration of extra-anatomic left subclavian bypass technique. Video available at: https://www.jtcvs.org/article/S2666-2507(25)00064-1/fulltext.
