# Supplementary figures and images for: Extra-anatomic left subclavian artery bypass patency in frozen elephant trunk surgery
Source: JTCVS Tech. 2025 Feb 11;30:1–7. doi: 10.1016/j.xjtc.2025.01.025 (PMC11998318; doi:10.1016/j.xjtc.2025.01.025)

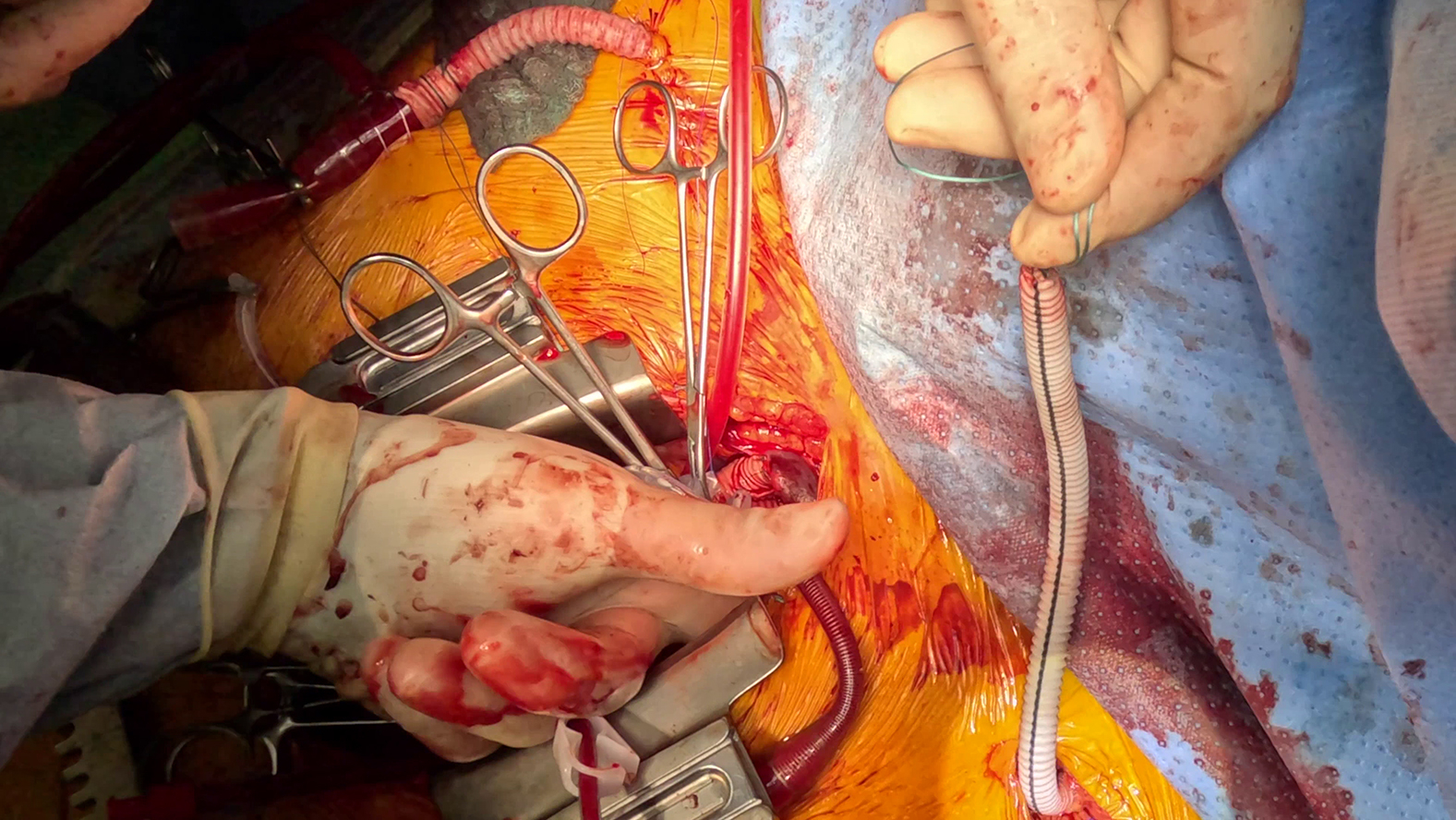

Supplement: Video 1 — Demonstration of extra-anatomic left subclavian bypass technique. Video available at: https://www.jtcvs.org/article/S2666-2507(25)00064-1/fulltext. [file fx2.jpg]
